# By what name shall I call thee?

**DOI:** 10.1371/journal.pgen.1009167

**Published:** 2020-10-29

**Authors:** Gregory S. Barsh, Gregory P. Copenhaver

**Affiliations:** 1 HudsonAlpha Institute for Biotechnology, Huntsville, AL, United States of America; 2 Department of Genetics, Stanford University School of Medicine, Stanford, CA, United States of America; 3 Department of Biology and the Integrative Program for Biological and Genome Sciences, University of North Carolina at Chapel Hill, Chapel Hill, NC, United States of America

**Fig 1:**
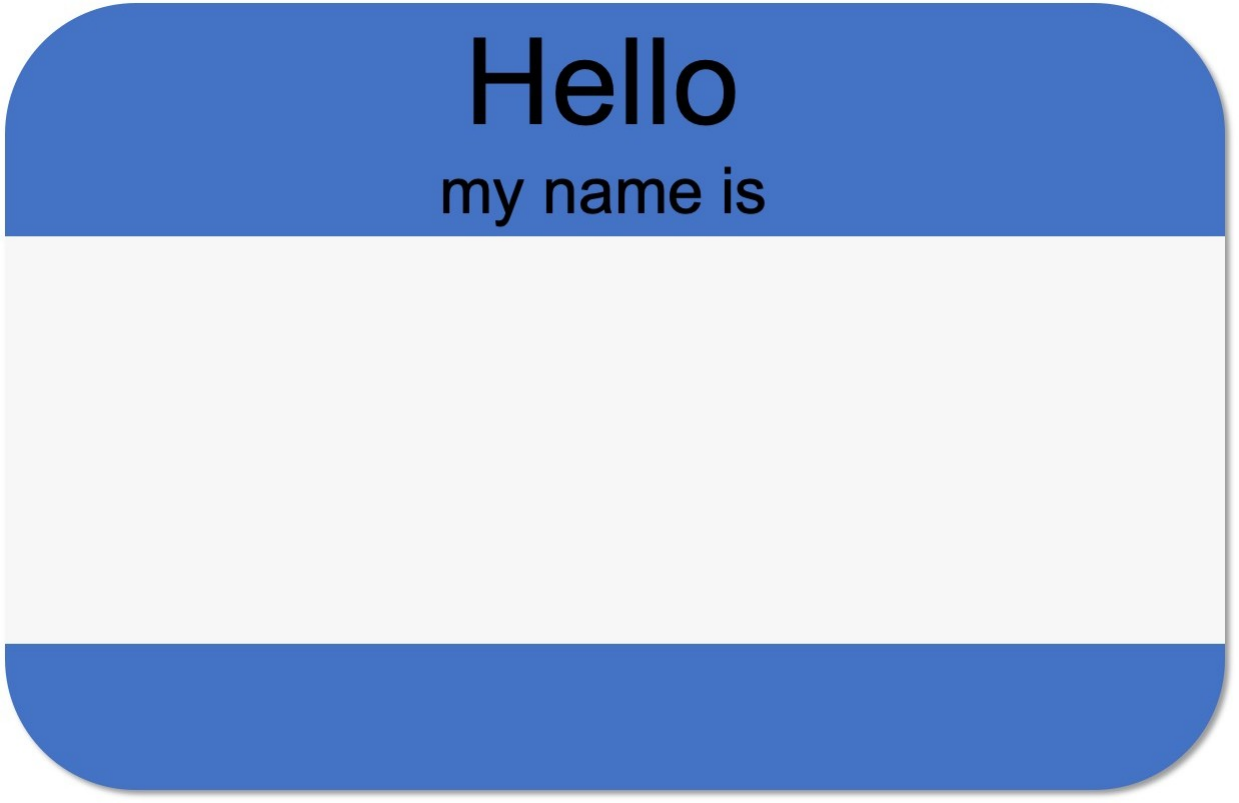
*PLOS Genetics* supports transgender and nonbinary authors and the use of their true names.

A scientist’s career, represented by their publication record, may span half a century or more. Facilitated by name-based search engines, it’s straightforward to ascertain, for example, that we began working on citrus DNA markers and phosphate uptake in fibroblasts, moved on to investigate the structure of centromeres and human collagen diseases, and now study meiotic recombination and color variation in mammals. Unique Open Researcher and Contributor ID (ORCID) identifiers enable the same task when a scientist changes their name (Fig 1) or if the same name is shared by different scientists. The ability to ascertain our, or any scientist’s, body of work assists evaluation of productivity, innovation, and the importance of individual contributions to respective fields and facilitates career progression and advancement. This process also creates a public record of name changes, commonly through marriage or divorce. In many instances, this record carries no more weight than a change of scientific direction. But in some cases, prior names and a public record of their change can be damaging or stigmatizing, particularly for transgender scientists.

Many transgendered scientists must continually ask that their name be recognized and appropriately connected with the entirety of their scholarly work. Having to ask for that acknowledgment creates an inequitable burden, and for some, it can be psychologically painful. *PLOS Genetics*, together with all of the PLOS family of journals, is proud to announce that we now welcome transgender and nonbinary authors to update their names on the papers they have published with us.

Upon request by the corresponding author, relevant papers will be republished. Republishing fully replaces the paper online. Republishing will update the paper’s indexing metadata, which should be reflected in how it appears in indexing services such as PubMed, Web of Science, and Google Scholar. This process will enable full replacement of the author’s name and ensure that citation information such as the DOI for the paper remains the same. All previous citations to the paper will remain valid. To maintain the author’s privacy, we will not attach a notice of the name change to the republished paper.

*PLOS Genetics* is committed to equality and inclusion. By what name shall we call you? Your name.

